# Acute Liver Injury Caused by Cyclophosphamide in a Patient With Factor VIII Deficiency: A Rare Presentation

**DOI:** 10.7759/cureus.55717

**Published:** 2024-03-07

**Authors:** Kais Antonios, Aciel Shaheen, Priyata Dutta, Michael Fine

**Affiliations:** 1 Internal Medicine, Trinity Health Ann Arbor Hospital, Ann Arbor, USA; 2 Gastroenterology, Trinity Health Ann Arbor Hospital, Ann Arbor, USA; 3 Gastroenterology and Hepatology, Huron Gastroenterology Associates, Ypsilanti, USA

**Keywords:** recam score, rare clinical presentation, acute liver injury, cyclophosphamide, drug-induced liver injury (dili)

## Abstract

Because of the variety of drugs, herbal, and dietary supplements used in clinical practice. Drug-induced liver injury (DILI) has become an important and common cause of acute liver injury and failure. Many drugs associated with DILI have been identified, but there remains some uncertainty about others. Cyclophosphamide is a commonly used antineoplastic medication, and its association with DILI has been reported in animals and has been established in humans with the use of high-dose IV. Oral cyclophosphamide has not been clearly shown to cause acute liver injury, thus highlighting many of the unique aspects of this manuscript. Here, we report a case of cyclophosphamide-induced DILI with the aim to alert clinicians regarding this potential association.

## Introduction

Drug-induced liver injury (DILI) is an important adverse event that can range from mild elevation in liver enzymes to acute liver failure, transplantation, or death. Studies regarding the incidence of DILI in the United States have been limited, although a 2013 study in Iceland estimated its incidence at around 19.1 cases per 100,000 inhabitants [1]. 

Cyclophosphamide is an alkylating agent widely used for hematological and rheumatic diseases including, but not limited to, non-Hodgkin lymphoma, lupus nephritis, and systemic sclerosis. Some of its more common side effects include bone marrow suppression leading to neutropenia, hair loss, nausea, and vomiting, in addition to hemorrhagic cystitis. Long-term use of cyclophosphamide has been shown to increase the incidence of malignancies of the bladder, the hematopoietic system, and the skin [2].

While mild hepatotoxicity has been associated with cyclophosphamide use in the past, acute liver injury (ALI) secondary to cyclophosphamide use is not a well-known nor an expected side effect [3,4]. In this article, we report a case of a 77-year-old male who developed ALI after receiving cyclophosphamide for the treatment of acquired hemophilia A. In addition, we will discuss the possible mechanisms suggested in the limited literature available about this association and possible interventions to prevent this complication.

## Case presentation

A 77-year-old male with a past medical history significant for hypertension, coronary artery disease, and a recent diagnosis of acquired factor 8 inhibitor presented to the emergency department in November of 2022 at the instruction of his outpatient hematologist due to elevated liver enzymes. He had initially presented in October of 2022 with spontaneous extensive ecchymoses of the left upper extremity. He was evaluated by hematology, and due to a prolonged activated partial thromboplastin time (aPTT) of 70.8 seconds (reference range 25.1-36.5 seconds), a mixing study was ordered, which was notable for immediate correction at time 0 hours. With severely low factor 8 activity at 2%, there was a high clinical suspicion of acquired factor 8 inhibition, which was confirmed with additional testing. The patient was discharged after being started on prednisone 120 mg daily, in addition to dapsone for pneumocystis carinii prophylaxis. His liver enzymes remained within normal limits during that admission.

**Figure 1:**
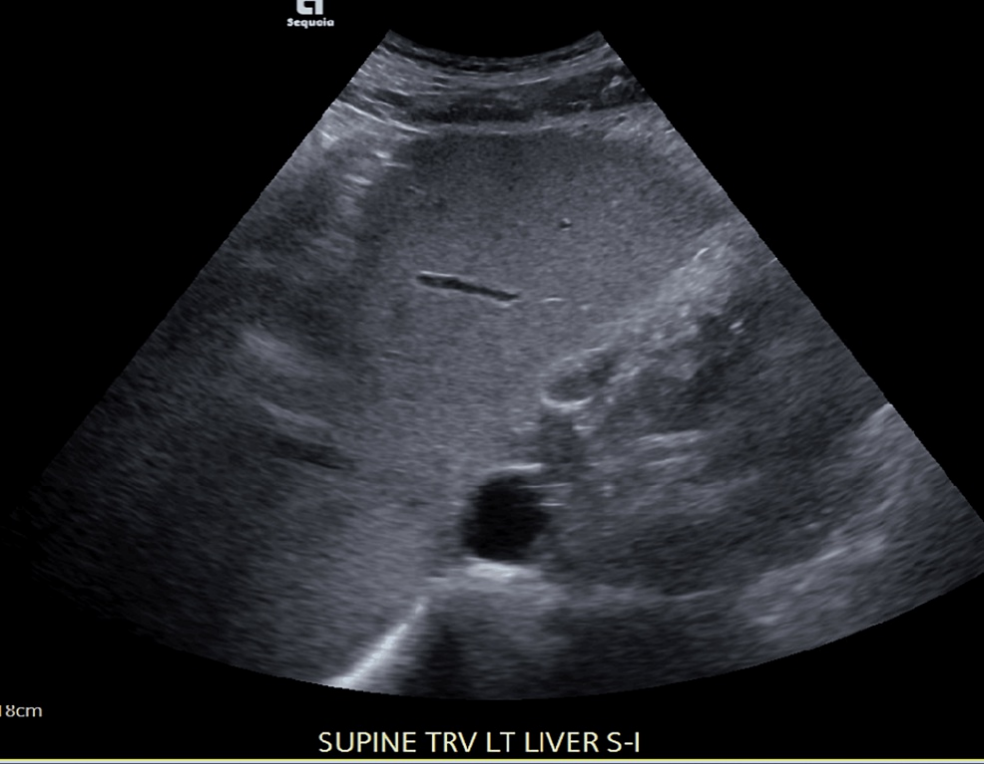
Ultrasound of the abdomen demonstrating a normal liver morphology

On follow-up with hematology, the patient was started on PO cyclophosphamide 200 mg daily (2 mg/kg), and in addition, he was slowly tapered off prednisone over one month. Eventually, dapsone was stopped as his prednisone dosage was reduced. One week after, on routine blood work, he was found to have an elevation of his aspartate aminotransferase (AST) and alanine aminotransferase (ALT) with an R factor of 13.5. Due to this elevation, he was instructed to stop taking cyclophosphamide the next day. On repeat blood work days after, his AST and ALT continued to increase, and he was instructed to present to the emergency department. He was admitted to the internal medicine service and remained overall asymptomatic during admission except for mild fatigue. International normalized ratio (INR), bilirubin, platelets, and alkaline phosphatase (ALP) remained within normal limits throughout his admission. Workup for other causes of hepatitis including viral, autoimmune, ischemic, and metabolic were all negative, and an ultrasound of the abdomen was unremarkable (Figure 1).

The highest concern was for DILI. A liver biopsy was considered, but AST and ALT started trending down significantly over the next few days, with ALT and AST values peaking on the second day of admission. Liver enzymes normalized completely one month after the initial injury, and cyclophosphamide was permanently discontinued. The pattern of the patient’s liver enzyme testing is best demonstrated in Table 1.

**Table 1:** Liver enzyme tests on pertinent days during the patient’s clinical course. RR: Reference range. All values are in unit/l. Day 0: Date of initiating cyclophosphamide therapy. Day 20: Day of stopping dapsone therapy. Day 26: Date of the first elevated liver enzyme test. Day 30: Date of hospital admission. Date 31: Date of peak elevation in liver enzymes. Date 60: Date of complete normalization in liver enzyme tests.

Lab value	AST (RR 13-39 unit/l)	ALT (RR 7-52 unit/l)	ALP (RR 27-120 unit/l)
Date			
Day 0	14	16	56
Day 20	20	25	53
Day 26	165	465	65
Day 30	1216	3605	79
Day 31	1443	3769	82
Day 60	20	36	55

## Discussion

Multiple drugs have been linked to DILI, and these drugs can precipitate DILI either in a predictable (dose-dependent or intrinsic) manner or an idiosyncratic manner. The most common of these drugs are listed in Table 2 [5].

**Table 2:** Drugs associated with DILI. This table was published by the European Association of the Study of the Liver clinical practice guidelines for DILI in the Journal of Hepatology, 2019-06-01, Volume 70, Issue 6, pages 1222-1261. For all open-access content, the Creative Commons licensing terms apply [5].

Intrinsic	Idiosyncratic	
Acetaminophen	Allopurinol	Lapatinib
Amiodarone	Amiodarone	Methyldopa
Anabolic steroids	Amoxicillin-clavulanate	Minocycline
Antimetabolites	Bosentan	Nitrofurantoin
Cholestyramine	Dantrolene	Pazopanib
Cyclosporine	Diclofenac	Phenytoin
Valproic acid	Disulfram	Pyrazinamide
HAART drugs	Felbamate	Propylthiouracil
Heparin	Fenofibrate	Statins
Nicotinic acid	Flucloxacillin	Sulfonamides
Statin	Flutamide	Terbinafine
Tacrine	Halothane	Ticlopidine
	Isoniazid	Tolvaptan
	Ketoconazole	Tolcapone
	Leflunomide	Trovafloxacin
	Lisinopril	

Cyclophosphamide is an extensively used anticancer and immunosuppressive agent. It is a prodrug that undergoes a complicated process of metabolic activation and inactivation. The acute toxicities of cyclophosphamide are related to its cytotoxicity. Like other alkylating agents, cyclophosphamide is most toxic to rapidly proliferating tissue, such as the hematopoietic system, epithelial cells of the gastrointestinal tract, hair follicles, and gonads [6]. Therefore, as mentioned above, common toxicities in cyclophosphamide treatment are nausea, vomiting, alopecia, immunosuppression, and gonadal damage.

Although the exact pathophysiology of cyclophosphamide-induced liver injury is yet to be clearly understood, it is thought to be related to acrolein, which is a highly active aldehyde formed in the degradation of cyclophosphamide metabolites that may enhance cyclophosphamide-induced cell damage, possibly by depletion of cellular glutathione by conjugation [7].

The dose at which cyclophosphamide causes liver injury has been variable, but it is generally considered a dose-dependent toxicity. Hepatotoxicity could be expected at doses delivered for preparation for bone marrow transplantation (2-4 g/m^2^), whereas at immunosuppressive doses (500-1000 mg/m^2^/once a month), hepatotoxicity is not a commonly anticipated side effect, although it has been previously been reported at 100 mg/day dosing [6,8]. In our case, the dose of cyclophosphamide was 200 mg/day. Although our patient was on two other medications that are associated with hepatotoxicity, namely, dapsone and prednisone, his ALI recovered while he was still taking prednisone, and despite cyclophosphamide and dapsone both having a high total DILI RECAM score of 11, which makes their association with this patient’s ALI very likely [9]. Dapsone had been discontinued before he developed the ALI, and the timeline of his ALI and recovery fit best with cyclophosphamide as the causative agent.

The exact period between initiating cyclophosphamide and developing hepatotoxicity varies from 24 hours to 24 months depending on the dose and route of administration [8,10]. However, the outcome of liver injury depends on how early drug cessation occurs. While most cases of hepatotoxicity should resolve after cessation of the particular drug, irreversible fulminant hepatic failure and even death have also been reported [10,11].

There have been some animal studies regarding experimental drugs or vitamins that could prevent the hepatic injury caused by cyclophosphamide. In a study performed on 40 mice by ElKhouly et al. [12], it was suggested that lutein, a carotenoid not produced by the human body and usually present in fruits and vegetables, has a potent protective role against cyclophosphamide-induced pulmonary and hepatic toxicities. This protective effect results from its ability to inhibit oxidative and nitrosative stress and to restore the activity of antioxidant enzymes and its ability to suppress lipid peroxidation. Another study performed on rats demonstrated a promising effect of apogliptin, which is a dipeptidyl peptidase 4 (DDP-IV) inhibitor mainly used as an antidiabetic agent, in ameliorating chemotherapy-induced liver toxicity via tackling the SIRT1/FoxO1 and the PI3k/Akt pathways, resulting in abridged oxidative stress, apoptosis, and hepatocellular injury [13]. Similar results were shown using melatonin in mice [14].

## Conclusions

Cyclophosphamide is a medication that has shown its potential hepatotoxicity in both animals and humans. As in most DILIs, early diagnosis and implementing early intervention, like in our case, can reduce the significant risk of mortality and morbidity. Due to the rarity of this association between cyclophosphamide and DILI, it might not be feasible to create a specific antidote, although there are promising animal studies regarding supplements or drugs that can prevent it.
